# Pressure-Regulated Volume Control Ventilation Versus Pressure Control Ventilation on Oxygenation and Lung Dynamics of Neonates With Acute Respiratory Failure: A Quasi-experimental Study

**DOI:** 10.7759/cureus.88924

**Published:** 2025-07-28

**Authors:** Rounak Jahan, Mohammad Monir Hossain, Md Hasibuzzaman, Shahadat H Polash, Shovon Saha

**Affiliations:** 1 Pediatrics Department, Bangladesh Shishu Hospital and Institute, Dhaka, BGD; 2 Neonatal Medicine Department and Neonatal Intensive Care Unit (NICU), Bangladesh Shishu Hospital and Institute, Dhaka, BGD; 3 Trauma, Orthopedic, and Spine Surgery Department, National Institute of Traumatology and Orthopaedic Rehabilitation, Dhaka, BGD; 4 Critical Care Medicine Department, Dhaka Medical College Hospital, Dhaka, BGD; 5 Pediatrics and Child Health Department, Bangladesh Shishu Hospital and Institute, Dhaka, BGD

**Keywords:** acute respiratory failure, lung dynamics, mechanical ventilation in neonates, neonatal ventilation, oxygenation, pressure control ventilation, pressure-regulated volume control ventilation, respiratory failure in neonates, ventilator-induced lung injury

## Abstract

Introduction

Acute respiratory failure in neonates presents as a critical challenge in neonatal intensive care units (NICUs), where mechanical ventilation plays a key role in management. Pressure control ventilation (PCV) is a conventional mode commonly used for ventilatory support. With technological advancements, newer modes such as pressure-regulated volume control (PRVC) have emerged, offering some potential benefits. However, studies comparing the effectiveness of PRVC to PCV in improving oxygenation and lung dynamics in neonates remain limited. This study aims to compare the effects of the PRVC mode to the PCV mode of ventilation in neonates with acute respiratory failure.

Objective

The objective of this study is to evaluate and compare the effectiveness of PRVC and PCV ventilation modes on oxygenation and lung dynamics in neonates with acute respiratory failure. We hypothesized that the PRVC mode would result in better oxygenation and lung dynamics compared to PCV in neonates with acute respiratory failure.

Method

This quasi-experimental study was conducted at the NICU of Bangladesh Shishu Hospital and Institute (BSH&I) from April 2023 to March 2025. A total of 60 neonates meeting the inclusion and exclusion criteria were enrolled and nonrandomly allocated into two groups: PRVC (n=30) and PCV (n=30). Oxygenation parameters (partial pressure of oxygen {PaO₂}, peripheral oxygen saturation {SpO₂}, fraction of inspired oxygen {FiO₂}, and PaO₂/FiO₂ {P/F} ratio), lung dynamics (compliance, driving pressure, and respiratory rate {RR}), arterial blood gas (ABG) values (pH, partial pressure of carbon dioxide {PaCO₂}, and bicarbonate {HCO₃}), and ventilator parameters (tidal volume (V_T_), peak inspiratory pressure (PIP), positive end expiratory pressure {PEEP}, and mean airway pressure {MAP}) were assessed and compared at initiation and one hour and 24 hours post-ventilation between the two groups. Statistical analysis determined significant differences between groups.

Results

Baseline characteristics were similar between the two groups. At one hour, SpO₂ (p=0.002), PaO₂ (p=0.002), and the P/F ratio (p=0.001) were significantly higher in the PRVC group; this trend also persisted at 24 hours (p<0.05). FiO₂ requirements were lower in PRVC at one hour (p=0.036) and 24 hours (p=0.024). PRVC also resulted in a significantly lower respiratory rate at 24 hours (p=0.033). Tidal volume remained higher (p<0.05), while peak inspiratory pressure and mean airway pressure were consistently lower in the PRVC group (p<0.001). No significant differences were observed in lung compliance, driving pressure, PEEP, and ABG parameters within 24 hours.

Conclusion

This study concluded that the PRVC mode provides better oxygenation than the PCV mode with a lower mean airway pressure, while both modes have similar effects on lung dynamics in neonates with acute respiratory failure.

## Introduction

In neonatal critical care, mechanical ventilation is a crucial component of management for critically ill neonates, though it carries significant risks including ventilator-induced lung injury (VILI) [1,2]. Worldwide, 30%-75% of mechanically ventilated preterm neonates suffer from bronchopulmonary dysplasia (BPD), which is mostly caused by VILI [3].

To reduce these harmful effects, various ventilation modes have been developed and adopted in clinical practice. Worldwide, common ventilation modes in pediatric and neonatal intensive care units (NICUs) include volume control ventilation (VCV), pressure control ventilation (PCV), synchronized intermittent mandatory ventilation (SIMV), pressure support ventilation (PSV), high-frequency oscillatory ventilation (HFOV), neurally adjusted ventilatory assist (NAVA), and pressure-regulated volume control (PRVC) ventilation. Despite the availability of various ventilator modes, the choice of their use in neonates is typically guided by the experience of the intensive care physician and institutional protocols [4,5].

In volume control ventilation (VCV), a set tidal volume (V_T_) is delivered, and the peak inspiratory pressure (PIP) varies as needed to achieve this volume. By contrast, in pressure control ventilation (PCV), a set PIP is maintained, and the delivered V_T_ fluctuates based on the compliance and resistance of the patient’s thorax and lungs [6]. Pressure control ventilation modes (assist control or SIMV) are frequently preferred modes in pediatric mechanical ventilation practice [7]. Here, at a set pressure, in a stiff lung, the delivered tidal volume will be lower. The same amount of pressure will deliver a larger tidal volume when compliance improves. There is a potential risk for overdistension of the lungs when compliance improves during the ongoing treatment. At these times, clinical decision-making and ventilator adjustment are required [8]. Tidal volume rather than the peak inspiratory pressure is now recognized as the main determinant of VILI [9].

Pressure-regulated volume control (PRVC) is a kind of dual-control ventilation mode that avoids both the peak airway pressure variations of VCV and also the tidal volume variation of PCV. Here, tidal volume is used as a feedback control, and the pressure limit is adjusted continuously. This may provide good patient-ventilator synchrony, effective pressure support, adequate gas exchange, and limited ventilator-induced lung injury [10]. The flow pattern is also variable, which helps to deliver the same amount of tidal volume with lower peak pressures in PRVC [7].

There is a paucity of studies comparing PCV to PRVC modes in neonates. But several studies have been done in children and in adults showing PRVC to be superior to other conventional ventilator modes such as PCV and VCV [1,7].

Sachdev et al. (2005) demonstrated that PRVC significantly improved partial pressure of oxygen (PaO₂), PaO₂/fraction of inspired oxygen (FiO₂) (P/F) ratio, and respiratory index (RI) in children, particularly during the early stages of ventilation, suggesting better oxygenation [1]. The PRVC mode appeared to be better in ventilating adult chronic obstructive pulmonary disease (COPD) patients with acute exacerbations, generating a significantly low peak inspiratory pressure and more effective ventilation [10]. In adult patients undergoing lung-protective ventilation, a significant reduction in tidal volume and peak pressures was found in the PRVC group [11]. Another study found that the PRVC mode of mechanical ventilation is preferable in acute respiratory failure as it resulted in improved oxygenation at lower inflation pressures [12].

Despite the widespread use of mechanical ventilation in NICUs, the knowledge of the safety and efficacy of different ventilator modes is very limited, as there is a scarcity of evidence-based comparative studies between different ventilator modes. This study aimed to compare the pressure-regulated volume control (PRVC) mode to the pressure control ventilation (PCV) mode in improving oxygenation and lung dynamics of neonates with acute respiratory failure.

## Materials and methods

This study was conducted on neonates with acute respiratory failure admitted to the NICU of Bangladesh Shishu Hospital and Institute (BSH&I), following ethical approval from the Ethical Review Committee (ERC) of Bangladesh Shishu Hospital and Institute (approval number: Admin/BSHI/2023/916/1) and informed written consent from parents or legal guardians after explaining the study’s purpose, procedures, benefits, and potential risks.

Neonates between 34 and 40 weeks of gestational age presenting with acute respiratory failure were enrolled in this quasi-experimental study conducted from April 2023 to March 2025. Enrollment was based on clinical presentation, arterial blood gas (ABG) analysis, suspected etiology, and chest X-ray findings. The participants were assigned alternately into two groups: Group A (pressure control ventilation, PCV) and Group B (pressure-regulated volume control, PRVC), with the first neonate allocated by lottery. This alternate assignment method, though systematic, may introduce allocation bias due to a lack of true randomization. Detailed maternal history and clinical examination were documented for all neonates. Gestational age was determined using the New Ballard scoring system, last menstrual period (LMP), and corroborating antenatal records. Surfactant can influence lung compliance and oxygenation. To control for this variable, neonates who received surfactant were excluded before enrollment.

Vital signs, including respiratory rate (RR), heart rate, and peripheral oxygen saturation (SpO₂), were continuously monitored using a multiparameter monitor (via Edan X12 monitor, Edan Instruments, Inc., Shenzhen, China). Intubation was performed under aseptic conditions using an appropriate laryngoscope and endotracheal (ET) tube sizes. All patients were ventilated using the Tecme Neumovent GraphNet Ts ventilator (Atlanta, GA). PCV mode settings included PIP of 15-20 cmH₂O (preterm) or 20-25 cmH₂O (term), positive end expiratory pressure (PEEP) of 5-8 cmH₂O, RR 40-60 beats per minute (bpm), inspiratory time (Ti) of 0.4 seconds, and FiO₂ of 0.6-0.75. PRVC mode settings included V_T_ of 4-6 mL/kg (preterm) or 5-7 mL/kg (term), PEEP of 5-8 cmH₂O, RR of 40-60 bpm, Ti of 0.4 seconds, FiO₂ of 0.6-0.75, and maximum pressure limit of 30 cmH₂O. The ventilation goal was to maintain ABG within a pH of 7.25-7.45, PaO₂ of 50-70 mmHg, partial pressure of carbon dioxide (PaCO₂) of 35-55 mmHg, and SpO₂ of 90%-95%. Continuous monitoring of SpO₂ and heart rate, along with the clinical assessment of respiratory status and perfusion, guided the ventilator adjustments. ABG data were recorded at initiation, one hour, and 24 hours post-ventilation using 1 mL arterial blood analyzed by Siemens RAPIDPoint 500 (Manchester, United Kingdom) within 15 minutes of collection. The ET tube position was confirmed with a portable chest X-ray after intubation and by auscultation every four hours. Gentle suctioning of the ET tube was done as needed. Lung compliance was assessed via an inspiratory hold maneuver at all three time points. Driving pressure was calculated as PIP, PEEP in PCV mode and plateau pressure (pPlat), and PEEP in PRVC mode. Ventilator settings in PCV were reduced by gradually lowering FiO₂, PIP, RR, and PEEP to maintain target oxygenation and ventilation. In PRVC, settings were weaned by adjusting FiO₂, RR, and PEEP, with PIP regulated automatically by the ventilator. While individualized care is essential in neonatology, we acknowledge that this introduces a degree of subjectivity.

**Figure 1:**
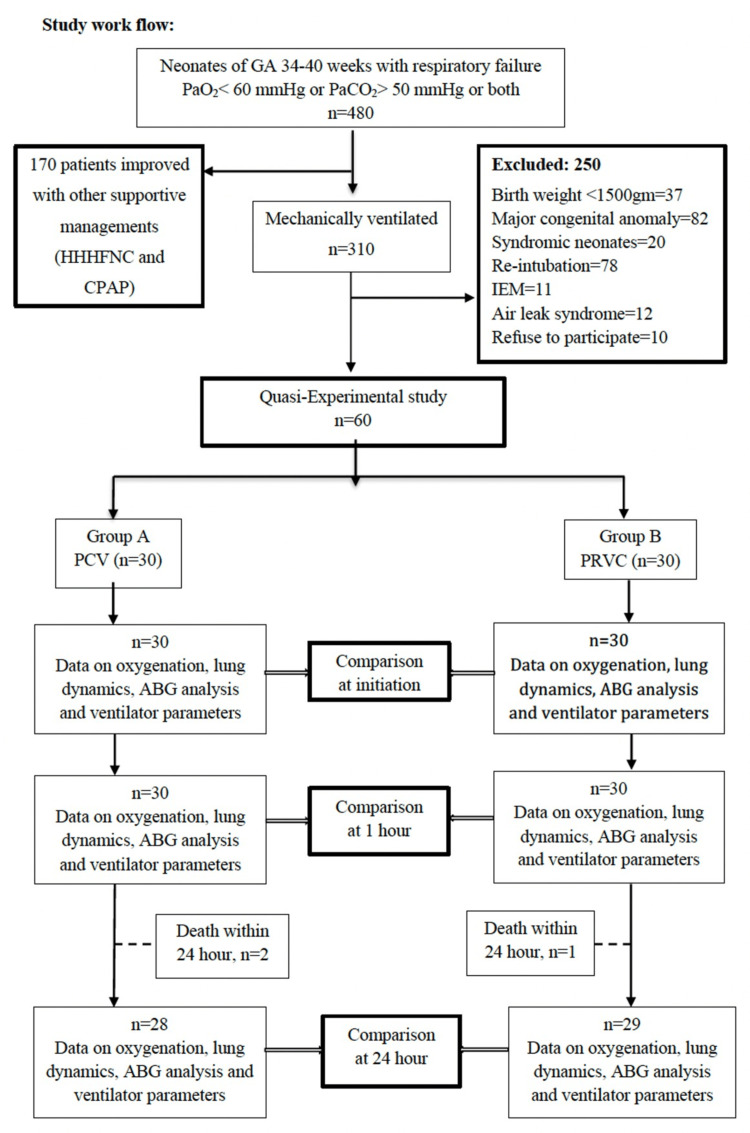
Study flow diagram GA, gestational age; PaO₂, partial pressure of oxygen; PaCO₂, partial pressure of carbon dioxide; HHHFNC, heated humidified high-flow nasal cannula; CPAP, continuous positive airway pressure; IEM, inborn error of metabolism; PCV, pressure control ventilation; PRVC, pressure-regulated volume control; ABG, arterial blood gas

The final analysis compared oxygenation parameters, lung dynamics, ABG results, and ventilator settings across both groups at the defined time points. After 24 hours, patients continued ventilation per NICU protocols (Figure 1).

Statistical analysis

Following data collection, the data were manually reviewed, revised, and validated before being tabulated in order to ensure consistency. Computers were used to code, enter, and analyze data by the Statistical Package for Social Sciences (SPSS, version 26.0) (IBM Corp., Armonk, NY). Data were expressed as numbers and percentages for categorical variables or median (interquartile range) for quantitative variables. To compare categorical variables, the chi-square (χ^2^) test and Fisher’s exact test were used. Quantitative variables were compared using the Mann-Whitney U test. The results of the statistical analysis were presented in tables. For all statistical tests, a p value of <0.05 was considered statistically significant.

## Results

Finally, a total of 57 neonates out of 60 completed this study. Among them, 28 were in the PCV group, and 29 were in the PRVC group.

Table 1 shows that the median age at ventilation in the PCV and PRVC groups was five days and six days, respectively, and the difference was not statistically significant (P>0.05). The majority of the study participants of both groups were male neonates, but the distribution was statistically not different (P>0.05). In the PCV group (N=30), 12 (30, 40.0%) neonates had gestational age from 34 to <37 weeks and 18 (60.0%) from 37 to 40 weeks, while in the PRVC group (N=30), 11 (36.7%) neonates had gestational age from 34 to <37 weeks and 19 (63.3%) from 37 to 40 weeks. The differences were not statistically significant (P>0.05). The majority of the patients in both groups were delivered through vaginal delivery, and the median birth weight of the patients was 2900 (2100, 3200) g and 2800 (2200, 3200) g in the PCV (N=30) and PRVC (N=30) groups, respectively. There was no significant statistical difference between the groups (P>0.05).

**Table 1:** Comparison of baseline characteristics between the two groups Data are expressed as median (interquartile range {IQR}) and number (percentage). For statistical analysis, the Mann-Whitney U test and chi-square test were done between the two groups (^a^Mann-Whitney U test; ^b^chi-square test). P<0.05=significant PCV, pressure control ventilation; PRVC, pressure-regulated volume control

Baseline characteristics	PCV (n=30)	PRVC (n=30)	U value/chi-square value (χ^2^)	P value
Age at ventilation (days)				
Median (IQR)	5.0 (3.0, 7.7)	6.0 (3.0, 9.0)	429.5	0.760^a^
Gender				
Male	22 (73.2%)	19 (63.3%)	0.693	0.405^b^
Female	8 (26.7%)	11 (36.7%)		
Mode of delivery				
Vaginal delivery	18 (60.0%)	21 (70.0%)	0.659	0.417^b^
Cesarean section	12 (40.0%)	9 (30.0%)		
Gestational age (weeks)				
34 to <37	12 (40.0%)	11 (36.7%)	0.071	0.791^b^
37 to 40	18 (60.0%)	19 (63.3%)		
Birth weight (g)				
Median (IQR)	2900 (2100, 3200)	2800 (2200, 3200)	417.5	0.630^a^

Table 2 shows that the majority of the patients in both groups had sepsis, 14 (46.7%) in the PCV group (N=30) whereas 10 (33.3%) in the PRVC group (N=30), without any significant difference (P>0.05). There was also no significant statistical difference found between the two groups regarding perinatal asphyxia, pneumonia (PNA), respiratory distress syndrome (RDS), and meconium aspiration syndrome (MAS) (p>0.05). As neonates experienced multiple types of diseases at the same time, column percentages do not add up to 100%.

**Table 2:** Comparison between two groups according to initial diagnosis Data are expressed as number (percentage). For statistical analysis, a chi-square test was done between the two groups (^a^chi-square test). P<0.05=significant PCV, pressure control ventilation; PRVC, pressure-regulated volume control

Initial diagnosis	PCV (n=30)	PRVC (n=30)	χ^2^	P value
Sepsis	14 (46.7%)	10 (33.3%)	1.111	0.292^a^
Perinatal asphyxia	13 (43.3%)	12 (40.0%)	0.069	0.793^a^
Pneumonia	9 (30.0%)	8 (26.7%)	0.082	0.774^a^
Respiratory distress syndrome	7 (23.3%)	8 (26.7%)	0.089	0.766^a^
Meconium aspiration syndrome	3 (10.0%)	2 (6.7%)	0.218	0.500^a^

Table 3 shows that, at initiation, the median SpO₂, PaO₂, FiO₂, and P/F ratio were statistically similar between the PCV (N=30) and PRVC groups (N=30) (p>0.05). After one hour, SpO₂ increased significantly in the PRVC group (93.0%) compared to the PCV group (91.0%), with both groups having equal sample sizes (N=30; p=0.002). This trend persisted at 24 hours, with SpO₂ remaining higher in the PRVC group (96.0%) compared to the PCV group (95.0%), with both groups having equal sample sizes (N=30; p=0.034). Similarly, after one hour, PaO₂ increased significantly in the PRVC group (112.8 mmHg) compared to the PCV group (98.8 mmHg) (N=30; p=0.002). By 24 hours, PaO₂ remained significantly higher in the PRVC group (135.4 mmHg) than in the PCV group (126.6 mmHg) (p=0.019). After one hour, FiO₂ decreased significantly in the PRVC group (60%) compared to the PCV group (65%) (N=30; p=0.036). This reduction persisted at 24 hours, with FiO₂ lower in the PRVC group (50%) than in the PCV group (55%) (p=0.024). For the P/F ratio, after one hour, it increased significantly in the PRVC group (185.7) compared to the PCV group (154.9) (N=30; p=0.001). By 24 hours, the P/F ratio remained significantly higher in the PRVC group (251.9) than in the PCV group (232.0) (N=30; p=0.010).

**Table 3:** Comparison of oxygenation parameters in the two groups (n=60) Data are expressed as median (interquartile range). For statistical analysis, the Mann-Whitney U test was done, and P<0.05=significant SPO₂, peripheral oxygen saturation; PaO₂, partial pressure of oxygen; FiO₂, fraction of inspired oxygen; P/F, PaO₂/FiO₂; PCV, pressure control ventilation; PRVC, pressure-regulated volume control

Oxygenation parameters	PCV	PRVC	U value	P value
SPO₂ (%)				
At initiation (n=30, 30)	87.0 (83.0, 88.2)	86.0 (82.0, 87.2)	396.0	0.423
After one hour (n=30, 30)	91.0 (90.0, 92.0)	93.0 (92.0, 94.0)	240.5	0.002
After 24 hours (n=28, 29)	95.0 (94.0, 96.0)	96.0 (95.0, 96.0)	280.0	0.034
PaO₂ (mmHg)				
At initiation (n=30, 30)	56.2 (50.8, 58.6)	55.0 (51.2, 57.8)	436.5	0.842
After one hour (n=30, 30)	98.8 (89.4, 110.6)	112.8 (107.0, 120.4)	246.5	0.002
After 24 hours (n=28, 29)	126.6 (112.0, 136.4)	135.4 (126.4, 140.2)	261.5	0.019
FiO₂​​​​​​​ (%)				
At initiation (n=30, 30)	70.0 (63.7, 71.8)	70.0 (63.7, 71.2)	435.5	0.826
After one hour (n=30, 30)	65.0 (60.0, 70.0)	60.0 (55.0, 65.0)	311.0	0.036
After 24 hours (n=28, 29)	55.0 (50.0, 60.0)	50.0 (45.0, 55.0)	249.0	0.024
P/F ratio				
At initiation (n=30, 30)	80.6 (72.3, 88.8)	79.2 (71.3, 99.2)	449.5	0.994
After one hour (n=30, 30)	154.9 (132.7, 182.8)	185.7 (170.9, 212.2)	228.5	0.001
After 24 hours (n=28, 29)	232.0 (204.0, 243.0)	251.9 (231.0, 266.3)	225.0	0.010

Table 4 shows that the lung dynamic parameters, including respiratory rate, compliance, and driving pressure, were comparable between the two groups (PCV and PRVC; N=30 each) at initiation and after one hour of ventilation (p>0.05). However, after 24 hours, the PRVC group demonstrated a statistically significant reduction in respiratory rate (p=0.033), while compliance and driving pressure remained unchanged between the groups (p>0.05).

**Table 4:** Comparison of lung dynamics between the two groups Data are expressed as median (interquartile range). For statistical analysis, the Mann-Whitney U test was done, and P<0.05=significant PCV, pressure control ventilation; PRVC, pressure-regulated volume control

Lung dynamics	PCV	PRVC	U value	P value
Respiratory rate (/minute)				
At initiation (n=30, 30)	65.0 (54.7, 68.0)	62.0 (52.0, 66.0)	368.0	0.222
After one hour (n=30, 30)	49.0 (46.0, 60.5)	48.0 (43.5, 52.5)	346.5	0.124
After 24 hours (n=28, 29)	42.0 (40.0, 42.0)	39.0 (36.0, 41.0)	263.0	0.033
Compliance (mL/cmH_2_O)				
At initiation (n=30, 30)	1.2 (1.1, 1.6)	1.2 (1.1, 1.6)	443.5	0.923
After one hour (n=30, 30)	1.5 (1.2, 1.9)	1.5 (1.4, 1.8)	414.5	0.559
After 24 hours (n=28, 29)	2.0 (1.7, 2.3)	2.0 (1.8, 2.6)	362.5	0.487
Driving pressure (cmH_2_O)				
At initiation (n=30, 30)	16.0 (14.0, 16.0)	15.0 (14.0, 16.0)	425.5	0.708
After one hour (n=30, 30)	14.5 (12.7, 16.0)	13.5 (12.0, 15.0)	349.0	0.131
After 24 hours (n=28, 29)	12.5 (11.0, 15.0)	12.0 (10.0, 14.0)	296.0	0.075

Table 5 presents the arterial blood gas (ABG) parameters of neonates in the PCV and PRVC groups (N=30 each). The median values of pH, PaCO₂, and bicarbonate (HCO₃) remained statistically similar between the groups at initiation, after one hour, and after 24 hours of ventilation (p>0.05). No significant differences were observed in any of these parameters over time.

**Table 5:** Comparison of arterial blood gas (ABG) analysis in two groups Data are expressed as median (interquartile range). For statistical analysis, the Mann-Whitney U test was done, and P<0.05=significant PCV, pressure control ventilation; PRVC, pressure-regulated volume control; PaCO₂, partial pressure of carbon dioxide; HCO₃, bicarbonate

ABG parameters	PCV	PRVC	U value	P value
pH				
At initiation (n=30, 30)	7.2 (7.1, 7.3)	7.2 (7.1, 7.3)	0.667	0.667
After one hour (n=30, 30)	7.3 (7.2, 7.4)	7.3 (7.2, 7.4)	0.842	0.842
After 24 hours (n=28, 29)	7.4 (7.3, 7.4)	7.4 (7.3, 7.4)	0.163	0.163
PaCO₂ (mmHg)				
At initiation (n=30, 30)	64.1 (51.3, 71.3)	65.3 (52.1, 69.6)	0.935	0.935
After one hour (n=30, 30)	56.5 (46.1, 60.1)	53.6 (47.3, 59.2)	0.433	0.433
After 24 hours (n=28, 29)	46.2 (40.7, 48.6)	43.2 (40.4, 47.7)	0.517	0.517
HCO₃​​​​​​​ (mmol/L)				
At initiation (n=30, 30)	22.9 (18.8, 25.7)	22.9 (19.0, 26.1)	0.848	0.848
After one hour (n=30, 30)	23.0 (21.8, 25.1)	23.6 (19.4, 24.8)	0.712	0.712
After 24 hours (n=28, 29)	24.0 (21.9, 24.4)	23.6 (22.3, 25.7)	0.980	0.980

Table 6 shows that, at initiation, the tidal volume was significantly higher in the PRVC group (N=30) compared to the PCV group (N=30) (p=0.041), a difference that persisted at both one hour (p=0.020) and 24 hours (p=0.015). Conversely, the PIP was significantly lower in the PRVC group compared to the PCV group at initiation (p=0.029) and remained so after one hour (p<0.001) and 24 hours (p<0.001). There was no statistically significant difference in PEEP between the two groups at any time point (p>0.05). Although MAP was similar between the groups at initiation (p>0.05), it became significantly lower in the PRVC group compared to the PCV group after one hour (p<0.001) and 24 hours (p<0.001).

**Table 6:** Comparison of ventilator parameters between the two groups Data are expressed as median (interquartile range). For statistical analysis, the Mann-Whitney U test was done, and P<0.05=significant PCV, pressure control ventilation; PRVC, pressure-regulated volume control; PIP, peak inspiratory pressure; PEEP, positive end expiratory pressure; MAP, mean airway pressure

Ventilation parameters	PCV	PRVC	U value	P value
Tidal volume				
At initiation (n=30, 30)	15.5 (12.0, 18.0)	18.0 (14.7, 21.0)	312.5	0.041
After one hour (n=30, 30)	15.5 (13.0, 18.0)	18.5 (15.0, 21.2)	293.5	0.020
After 24 hours (n=28, 29)	16.0 (15.0, 19.7)	19.0 (16.5, 22.0)	245.0	0.015
PIP (cmH_2_O)				
At initiation (n=30, 30)	22.0 (20.0, 22.0)	20.0 (19.0, 21.2)	308.5	0.029
After one hour (n=30, 30)	22.0 (20.0, 22.0)	18.5 (17.7, 20.0)	107.0	<0.001
After 24 hours (n=28, 29)	21.0 (20.0, 22.0)	18.0 (16.0, 18.0)	86.5	<0.001
PEEP (cmH_2_O)				
At initiation (n=30, 30)	6.0 (5.0, 6.0)	5.0 (5.0, 6.0)	446.5	0.950
After one hour (n=30, 30)	6.0 (5.0, 6.0)	5.0 (5.0, 6.0)	348.0	0.086
After 24 hours (n=28, 29)	5.0 (5.0, 5.7)	5.0 (5.0, 5.0)	358.5	0.268
MAP (cmH_2_O)				
At initiation (n=30, 30)	12.3 (11.9, 13.0)	12.1 (11.8, 12.9)	401.0	0.468
After one hour (n=30, 30)	12.0 (11.4, 12.8)	11.0 (10.6, 11.3)	114.5	<0.001
After 24 hours (n=28, 29)	11.1 (10.4, 11.7)	9.9 (9.2, 10.1)	111.0	<0.001

## Discussion

In this study, men were more in number than women, having 73.2% and 63.3% in PCV and PRVC groups, respectively, without any significant difference between the groups. It could be due to the tendency of parents to seek greater health care for their male child. ﻿This observation was similar with the findings of previous studies [1,7].

The results of this study showed that the median age at ventilation was five days in the PCV group and six days in the PRVC group, with no statistically significant difference between the two groups. This finding differs from the study by Al Mandhari et al. (2024), where most neonates were ventilated at a median age of one day [13]. This disparity may be attributed to differences in the underlying disease patterns in the current study. Here, the majority of neonates were term babies who required ventilation due to conditions such as PNA, sepsis, or both. These conditions typically necessitate mechanical ventilation later than lung pathologies such as pneumonia, RDS, or MAS.

In this study, a majority of the neonates of both the PCV and PRVC groups were from 37 to 40 weeks of gestation. The median birth weight of the neonates in the PCV group was 2900 (2100, 3200) g and in the PRVC group, it was 2800 (2200, 3200) g, and the difference was not statistically significant. This finding was not similar with previous studies where birth weight was lower in study neonates [13-15]. It may be due to their enrollment of more premature neonates.

The majority of neonates in the current study were diagnosed with sepsis and/or PNA, either as isolated conditions or in combination with other illnesses at the time of enrollment. This finding is consistent with the fact that sepsis and PNA are among the most common indications for mechanical ventilation in neonates [16].

PRVC likely improves oxygenation by ensuring consistent tidal volume and better alveolar recruitment [17-19]. Similarly, this study found a significant and sustained improvement in peripheral oxygen saturation (SpO₂) in neonates ventilated with PRVC compared to PCV mode (both at one hour and 24 hours of ventilation), consistent with findings by Jain et al. (2016) [20] and Polimeni et al. (2006) [18]. However, in some related articles, the authors reported no significant difference in SpO₂ between pressure control and volume control modes [7].

Partial pressure of oxygen (PaO₂) significantly improved after one hour and remained similar after 24 hours of ventilation in neonates on PRVC compared to PCV. This finding is consistent with studies by Sachdev et al. (2005) [1] and Yin et al. (2019) [21]. This may be due to PRVC’s ability to improve oxygenation by adjusting inspiratory pressure to maintain a stable tidal volume, preventing hypoventilation and atelectasis [1,2]. This mechanism enhances alveolar recruitment and ventilation-perfusion (V/Q) matching, leading to better gas exchange.

In the present study, FiO₂ in the PRVC mode was significantly reduced compared to the PCV mode at both one hour and 24 hours, consistent with findings by Jain et al. (2016) [20] and Kıhtır et al. (2022) [7]. This reduction is likely attributed to the consistent improvement in PaO₂ with the PRVC mode, enabling the operator to safely decrease FiO₂ levels. Additionally, PRVC enhances V/Q matching by ensuring adequate ventilation, leading to improved oxygenation and a more controlled FiO₂ reduction. However, in other studies, there was no significant difference in the rate of FiO₂ reduction between the two modes [2,6].

The P/F ratio showed significant improvement in neonates receiving PRVC ventilation compared to those on PC ventilation in this study, similar to the findings in other studies [1,21,22]. As the PaO₂ increases significantly and clinicians are able to reduce the FiO₂, the P/F ratio naturally improves. The decelerating flow in PRVC seems to improve oxygenation by enhancing alveolar inflation, prolonging gas exchange, and optimizing V/Q matching [1,22].

The respiratory rate at baseline and after one hour of ventilation was similar in both groups. However, it significantly decreased after 24 hours in the PRVC group, indicating an improvement in lung dynamics. Similarly, another study reported a lower respiratory rate in volume guarantee ventilation (VGV) (PRVC) [23]. This reduction might be due to PRVC’s ability to dynamically adjust inspiratory pressure to maintain a preset tidal volume, adapting to changes in lung compliance and resistance, thereby ensuring stable ventilation and respiratory rate. In contrast, PCV uses a fixed pressure without measuring lung compliance, which can lead to variable tidal volumes and respiratory rates [24]. However, another study observed that the respiratory rate in volume guarantee ventilation was similar to that in pressure control ventilation [20].

In the current study, lung compliance remained the same in both groups at initiation after one hour, and after 24 hours, indicating similar efficacy in the early stages of ventilation. However, some authors reported that volume guarantee ventilation (VGV) such as the PRVC mode significantly improves lung compliance compared to pressure control (PCV) and volume control (VCV) modes alone [22]. Additionally, in some other studies, it was demonstrated that VGV significantly reduces the incidence of chronic lung disease such as BPD in neonates, further supporting PRVC’s efficacy in reducing barotrauma and improving compliance [25]. Conversely, some authors found no significant difference in the incidence of BPD between PCV and VGV modes [13,14].

In this study, driving pressure remained similar between both modes over 24 hours. However, in a study on adult patients, it was seen that volume guarantee ventilation (PRVC) effectively minimized peak inspiratory pressure, thus reducing driving pressure. This finding suggests that adaptive ventilation modes such as PRVC may provide advantages in optimizing driving pressures and improving lung compliance [26].

The median pH levels of neonates ventilated with both PCV and PRVC modes were similar at the beginning, and this similarity was maintained after one hour and 24 hours of ventilation. This indicates that both modes were equally effective in correcting blood gas abnormalities associated with disease pathology. Similar results were observed in studies by Işık et al. (2023) [15], Sachdev et al. (2005) [1], and Kıhtır et al. (2022) [7]. These findings suggest that both PCV and PRVC modes are capable of managing CO₂ levels and maintaining pH when ventilatory parameters are appropriately adjusted [15].

In this study, the partial pressure of carbon dioxide (PaCO₂) remained similar in both groups at initiation, after one hour, and after 24 hours. This finding is similar to other studies, suggesting both PCV and PRVC modes are effective in managing CO₂ levels [1,7,15]. However, Klingenberg et al. (2011) demonstrated that VGV modes, such as PRVC, are more effective at maintaining optimal CO₂ levels than pressure control modes by ensuring a consistent tidal volume [19,27]. Other studies have shown that volume guarantee ventilation leads to fewer fluctuations in CO₂ levels, reducing both hypocarbia and hypercarbia [27]. Fluctuations in CO₂ levels can impair cerebral blood flow over time, potentially leading to intraventricular hemorrhage, which may cause a sudden deterioration in ventilated neonates [28].

Serum bicarbonate (HCO₃) levels were comparable between PCV and PRVC groups at initiation and throughout the 24-hour period. Similar findings were reported by Işık et al. (2023) [15] and Kıhtır et al. (2022) [7] in their comparisons of these two modes. This similarity can be attributed to their comparable effectiveness in regulating arterial carbon dioxide (PaCO₂) levels. Maintaining PaCO₂ within a normal range is crucial for acid-base homeostasis, as CO₂ levels directly influence the bicarbonate buffering system. Therefore, both PCV and PRVC are effective in controlling PaCO₂, leading to stable serum bicarbonate levels [21].

In this study, tidal volume (V_T_) in the PRVC mode was higher than in the PCV mode at initiation, as the operator sets an adequate V_T_ in PRVC. However, pressure (PIP) is the key setting in PCV, and V_T_ is machine-generated. This difference remained consistent at one and 24 hours. Similarly, Polimeni et al. (2006) [18] and Shaarawy et al. (2016) [10] found higher V_T_ in PRVC than in PCV. PRVC adjusts PIP to achieve the target V_T_, while PCV delivers fixed PIP, causing V_T_ to vary with lung compliance and resistance [10]. In cases where lung mechanics remain stable, the pressure required in PRVC may match that in PCV, leading to similar V_T_, which was seen in some other studies [7,14].

Peak inspiratory pressure (PIP) is lower at the initiation of ventilation and remains lower after one hour and 24 hours in PRVC mode compared to PCV mode in this study. This finding can be related to several other studies [1,10,14]. PRVC is a hybrid ventilation mode that adjusts the pressure automatically to deliver a set V_T_ while using the lowest possible pressure. It creates decelerating airflow, which helps to reduce PIP. Meanwhile, PCV delivers breaths at a fixed pressure without measuring lung compliance. Here, if lungs become stiffer or more resistant, higher PIP may be needed to maintain the same V_T_ [6].

The present study observed that positive end expiratory pressure (PEEP) remained equal in both PCV and PRVC modes at initiation and after 24 hours of ventilation. Similarly, Chacko et al. (2015) also reported no difference in PEEP between the two modes [29]. However, findings by Klingenberg et al. (2011) [27] indicated higher PEEP during PRVC ventilation, while Kıhtır et al. (2022) [7] found lower PEEP in the PRVC mode compared to PCV. Since PEEP settings are typically clinician-determined, they can be adjusted to be the same in both modes. As a result, variations in PEEP between PCV and PRVC are generally caused by clinical decisions rather than intrinsic differences between the ventilation modes [21].

Mean airway pressure (MAP) was initially similar in both PCV and PRVC modes but became significantly higher in the PCV mode after one hour and 24 hours of ventilation. This finding aligns with several other studies [1,19]. In mechanical ventilation, MAP is determined by multiple factors, including PIP, PEEP, and the inspiratory/expiratory (I/E) ratio. An increase in PIP raises MAP since MAP represents the average airway pressure throughout the respiratory cycle [30]. This may explain the higher MAP observed in the PCV mode in this study. However, some studies [14] reported higher MAP in the PRVC mode compared to the PCV mode, while Shaarawy et al. (2016) [10] found no difference in MAP between the two modes. Higher MAP can cause barotrauma, while lower MAP can cause inadequate oxygenation in ventilated neonates. So, keeping the MAP at an optimum level is necessary to maintain oxygenation and ventilation [21].

One of the limitations of this study is that ventilator parameter adjustments were not strictly standardized and were guided by the clinical judgment of individual physicians, which may have introduced variability in outcomes. Although initial ventilator settings were consistent across groups, subsequent changes were individualized based on patient condition, potentially affecting reproducibility. Additionally, group allocation followed an alternate assignment method after an initial lottery, which may introduce selection bias compared to more robust randomization techniques. The study was also not blinded, which could affect the objectivity of outcome assessment. Furthermore, long-term follow-up was not performed, and thus, outcomes beyond the initial 24-hour period, such as the duration of ventilation, the incidence of complications, or late mortality, were not assessed.

## Conclusions

This study concluded that the PRVC mode provides better oxygenation than the PCV mode with lower mean airway pressure, while both modes have similar effects on lung dynamics in neonates with acute respiratory failure.

The PRVC mode can be a better alternative to conventional modes such as PCV, as it improves oxygenation by generating optimum airway pressure. To improve current knowledge, a future multicentered randomized controlled trial with a larger sample size should be conducted. Long-term follow-up on pulmonary outcomes, the duration of mechanical ventilation, the incidence of ventilator-associated lung injury, the length of ICU and hospital stay, and mortality rates should also be considered.
